# Immune monitoring after pediatric liver transplantation – the prospective ChilSFree cohort study

**DOI:** 10.1186/s12876-018-0795-x

**Published:** 2018-05-16

**Authors:** Imeke Goldschmidt, André Karch, Rafael Mikolajczyk, Frauke Mutschler, Norman Junge, Eva Doreen Pfister, Tamara Möhring, Lorenzo d’Antiga, Patrick McKiernan, Deirdre Kelly, Dominique Debray, Valérie McLin, Joanna Pawlowska, Loreto Hierro, Kerstin Daemen, Jana Keil, Christine Falk, Ulrich Baumann

**Affiliations:** 1 Division of Paediatric Gastroenterology and Hepatology, Department of Paediatric Liver, Kidney and Metabolic Diseases, Hannover Medical School, Carl-Neuberg-Strasse 1, 30625 Hannover, Germany; 2 Epidemiological and Statistical Methods Research Group, Helmholtz Centre for Infection Research, Inhoffenstr. 7, 38127 Braunschweig, Germany; 3 Paediatric Liver, GI and Transplantation, Ospedali Riuniti di Bergamo, Largo Barozzi 1, 24128 Bergamo, Italy; 4 Liver Unit, Birmingham Childrens Hospital, Steelhouse Lane, Birmingham, B4 6NH UK; 5 Pédiatre Hépatologue, Service d’Hépatologie-Gastroentérologie-Nutrition, Hôpital Necker-Enfants Malades, 149 rue de Sèvres, 75015 Paris, France; 6 Hopitaux Universitaires de Geneve, Hopital des Enfants pt Pédiatrie, Serv. Spécialités Pédiatriques, Rue Gabrielle-Perret-Gentil 4, 1211 Genève 4, Switzerland; 7 Centrum Zdrowia Dziecka, Al. Dzieci Polskich 20, 04-730 Warszawa, Poland; 8 Servicio de Hepatologia y Transplante, Hospital Infantil Universitario La Paz Madrid, Paseo de la Castellana 261, 28046 Madrid, Spain; 9 Institute of Transplant Immunology, IFB-Tx, Hannover Medical School, Car-Neuberg-Str. 1, 30625 Hannover, Germany; 10 Institute for Medical Epidemiology, Biometrics and Informatics, Martin-Luther-University Halle-Wittenberg, 06097 Halle (Saale), Germany; 11 Paediatric Hepatology Program, Children’s Hospital of Pittsburgh, One Children’s Hospital Way, 4401 Penn Ave, Pittsburgh, PA 15224 USA

**Keywords:** Paediatric liver transplantation, Immune monitoring, Rejection

## Abstract

**Background:**

Although trough levels of immunosuppressive drugs are largely used to monitor immunosuppressive therapy after solid organ transplantation, there is still no established tool that allows for a validated assessment of functional degree of immunosuppression or the identification of clinically relevant over- or under-immunosuppression, depending on graft homeostasis. Reliable non-invasive markers to predict biopsy proven acute rejection (BPAR) do not exist. Literature data suggest that longitudinal measurements of immune markers might be predictive of BPAR, but data in children are scarce. We therefore propose an observational prospective cohort study focusing on immune monitoring in children after liver transplantation. We aim to describe immune function in a cohort of children before and during the first year after liver transplantation and plan to investigate how the immune function profile is associated with clinical and laboratory findings.

**Methods:**

In an international multicenter prospective approach, children with end-stage liver disease who undergo liver transplantation are enrolled to the study and receive extensive immune monitoring before and at 1, 2, 3, 4 weeks and 3, 6, 12 months after transplantation, and whenever a clinically indicated liver biopsy is scheduled. Blood samples are analyzed for immune cell numbers and circulating levels of cytokines, chemokines and factors of angiogenesis reflecting immune cell activation. Statistical analysis will focus on the identification of trajectorial patterns of immune reactivity predictive for systemic non-inflammatory states, infectious complications or BPAR using joint modelling approaches.

**Discussion:**

The ChilSFree study will help to understand the immune response after pLTx in different states of infection or rejection. It may provide insight into response mechanisms eventually facilitating immune tolerance towards the graft. Our analysis may yield an applicable immune panel for non-invasive early detection of acute cellular rejection, with the prospect of individually tailoring immunosuppressive therapy. The international collaborative set-up of this study allows for an appropriate sample size which is otherwise difficult to achieve in the field of pediatric liver transplantation.

## Background

Successful immunosuppression (IS) in pediatric liver transplantation balances the degree of systemic immunosuppression between under- and over-suppression, minimizing the risks of graft rejection, infection and malignancy. The clinical challenge of how to best define, measure and monitor this optimal state has not been answered other than by clinical experience correlating a desired systemic non-inflammatory state to therapeutic drug monitoring [1].

Numerous approaches have been evaluated in order to monitor the effects of calcineurin inhibition and to guide the dosing of IS medication. An ideal monitoring tool would be easy to use in routine clinical care and yield rapid results that enable dose adjustments and a timely control of their effect. It would be cost-effective and would enable clinicians to navigate the fine individual line between just enough IS to prevent rejection, and as little IS as possible to minimize side effects. Current clinical practice predominantly uses pharmacokinetics, i.e. trough levels of immunosuppressive drugs, sometimes enhanced by C2 (2 h post dosing) levels and area under the curve (AUC) pharmacokinetics [2]. However, it is recognized that pharmacokinetic drug monitoring does not necessarily reflect the functional degree of immunosuppression. In studies that investigated IL-2 and IFN-γ production in T-cells as well as soluble IFN-γ, IL-2 and IL-17 concentrations as measures for the functional degree of immunosuppression, the degree of inhibition of cytokine production was not strongly associated with plasma tacrolimus or mycophenolic acid (MMF) concentrations [3]. Moreover, there was no difference in tacrolimus trough levels and tacrolimus AUC values or MMF AUC values between patients who did or did not reject [3, 4].

A number of approaches trying to describe the functional changes in the immune system after initiation of IS therapy have been published [3–7]. The main effect of calcineurin inhibitors is the inhibition of T cell activation by suppressing translocation of the NFAT transcription factor into the nucleus which blocks transcription and secretion of IL-2, GM-CSF and other cytokines from T cells. Therefore, the majority of experimental approaches focus on T cell activation, proliferation and cytokine secretion. Reduced T cell activation has been demonstrated in comparisons of adult kidney, heart and liver transplant recipients with either healthy adults [5] or intra-individually with pre and post dosing studies in transplanted patients 2 h after Cyclosporin A (CSA) ingestion compared to baseline [6, 7].

The measurement of circulating cytokine levels in the peripheral blood plasma represents an indirect measure of T cell activation, but allows assessing both TH1, TH2 and TH17 patterns at the same time. It also permits to analyze the complex interplay and cross-activation of different immune cells. Studies that measured circulating levels of IFN-γ, TNF-α, IL-2, soluble IL-2 receptor (sCD25), IL-10, IL-4, IL5, IL-12-p70, IL-12p40 and IL-15 at various time points before and after transplantation demonstrated an increase of both TH1 and TH2 cytokines shortly after transplantation, with differences in cytokine levels thereafter reflecting the occurrence of biopsy-proven acute cellular rejection (BPAR) [8–11].

Several other studies have examined immune markers in a longitudinal fashion in order to investigate predictors of BPAR. Observation periods ranged from very short-term (before transplantion (Tx) until 1 week after Tx) to long-term (before Tx until 24 months after Tx). While the results with regards to an association of pre-transplant variables with the occurrence of early BPAR are variable [3, 4, 12, 13], most studies reported a strong association of early changes after transplantation with the occurrence of BPAR [3, 11, 12]. In a study that followed liver transplanted adults for up to 1 year after transplantation, IFN-γ and IL-2 intracellular staining in T-cells and soluble concentrations compared to baseline (“percentage of inhibition”) during first week post-transplant differed between rejectors and non-rejectors [3]. In another group of 47 adult liver transplant recipients, serum concentrations of IFN-γ and IL-12-p70/IL-12p40 (TH1 cytokines), IL-4, IL-6 and IL-10 (TH2 cytokines) and TGF-ß (TH3 cytokines) were analyzed pre-transplant, 12 h post-transplant, daily until day 15 and every 3 days from day 15 to 30. Both rejectors and non-rejectors showed an increase of cytokines early after transplantation (day 3–9); however, TH2 cytokines clearly dominated in non-rejectors whereas levels of TH1 and TH2 were similar with slight dominance of TH1 cytokines in rejectors [11].

The number of studies examining functional immune changes after solid organ transplantation in children is still limited. A significant increase of donor-specific Granzyme B ELISPOT frequencies at day seven post-transplant compared to baseline was linked to the occurrence of BPAR in 28 pediatric liver transplant recipients [12]. Gras et al. [8] performed a prospective analysis in 40 children after LTx, 8 of whom experienced early BPAR. Circulating cytokine levels at 1 and 2 h after reperfusion and on day 1, 4, 7, 14 and 28 after Tx were determined. IL-4 was found to be decreased compared to baseline at all time points in the acceptance group [8].

In a large study incorporating 105 liver transplanted children for cross-sectional analysis with a subgroup of 22 children for longitudinal analysis, circulating cytokine levels at 12 months post-transplant differed between rejectors and non-rejectors. In the longitudinal analysis, there was a marked change in soluble cytokine levels compared to baseline at 4 weeks after transplantation, but none thereafter [9]. In addition, there was a strong association of TH11 and TH12 cytokine levels with age that was independent of the occurrence of BPAR.

### Aims and objectives

A clear-cut biomarker panel that provides information on the functional degree of immunosuppression and that may allow individualized prediction of tolerance or the risks of rejection or infection has not been established. In pediatric medicine the respective roles of the innate and the adaptive immune system during growth and development and the background of heterogeneous inherited liver diseases have also not been addressed yet, and the privileged role of the liver in transplantation has not been fully understood. Previous studies suffer from small sample sizes, especially in the area of pediatric solid organ transplantation. The few longitudinal studies published suggest that it is the interplay of immune markers over time rather than an absolute value at any given time point that is associated with the occurrence of tolerance.

It is for these reasons that in 2012 a consortium of seven European pediatric liver transplant centers joined an enprEMA recognized network to create the observational ChilSFree study. ChilSFree monitors immune markers such as cytokine, chemokine and angiogenic protein levels as well as immune cell numbers in peripheral blood from before transplantation until 12 months after transplantation. Our study aims at characterising the features of the innate and adaptive immune system before transplantation and after the introduction of immunosuppressive therapy after liver transplantation over the course of time. It uses a prospective longitudinal approach in order to link changes in the observed immune markers to the occurrence of clinical events such as rejection or infection. The multicenter approach will allow for sufficient numbers of participants. The scientific aim is to define markers of immune functioning that are specific to the functional degree of immunosuppression after liver transplantation which could be tested for their use for clinical management of minimized drug induced immunosuppression in future interventional trials.

## Methods

### Study design

**Fig. 1:**
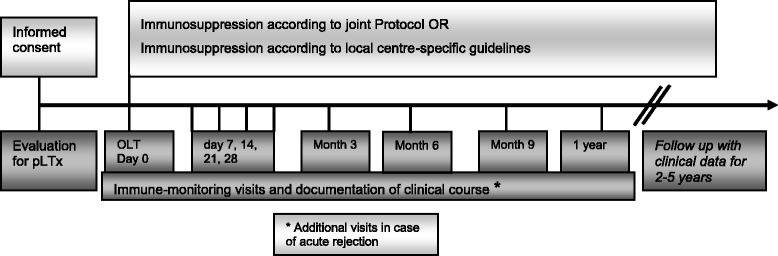
Study design

The study is designed as a prospective, observational cohort study. Children under the age of 18 years who receive de novo liver transplantation at one of seven participating European centers are considered for inclusion in the study; they undergo blood sampling for study purposes before transplantation and at 1, 2, 3, and 4 weeks as well as 3, 6 and 12 months after transplantation (Fig. 1). Additional samples are being obtained on the occasion of clinically suspected acute cellular rejection. Blood samples for analysis of immune markers are sent overnight to a central laboratory and are centrally analyzed. Clinical data is recorded locally in a purpose-built internet and Marvin-based electronic case report form (eCRF).

Immunosuppressive therapy will follow the local protocols and will be adjusted according to clinical needs as per the local protocols. The majority of the participating centres have previously been following very similar immunosuppressive protocols. Five of the participating centres use a standard steroid-free protocol for immunosuppression with Anti IL2R (Simulect; 10 mg intravenously if weight < 35 kg, 20 mg if > 35 kg at postoperative day (POD) 0 and POD4) and Tacrolimus (initially Modigraf, if and when appropriate followed by Prograf; loading dose: 0,1 mg/kg; then 0,05 mg/kg/12hrly; target ranges: week 1 and 2: about 12 (10 to 15) mg/l, week 3 and 4: about 10 (8 to 12) mg/l, month 1 to 3: about 8 (6 to 10) mg/l, month 4 to 12: about 6 (4 to 8) mg/l, thereafter about 4 (2 to 6) mg/l; if cellcept is added, target ranges will be 2 mg/l lower).

Deviations from the above protocol in all centres will be made as clinically indicated and will be documented in the eCRF. Children will undergo blood sampling for immune monitoring at the occasion of routine clinical visits. Neither additional visits nor additional blood samplings are planned. Medical therapy will be applied as clinically indicated and per the local post-transplantation protocols. No additional interventions are proposed by the observational ChilSFree study protocol.

### Study population

A priori, all patients under the age of 18 years undergoing de novo liver transplantation will be eligible for participation irrespective of primary disease. Exclusion criteria have been defined as underlying conditions that may interfere with the patient’s safety, compliance or study evaluation in the opinion of the local investigator and absence of informed consent of parents or legal guardians or the (adolescent) patient. Re-transplantation has also been defined as an exclusion criterion as the study also examines the changes in the immune system compared to the pre-transplant status. In patients who undergo re-transplantation, the pre-transplant-status is not IS naïve.

### Immune monitoring

The study will examine both cellular and humoral immune markers. Subsets of inflammatory cells (CD4+ and CD8+ T cells, CD19+ B cells, CD56/16 + NK cells, CD56+ T cells, monocytes and granulocytes) will be measured using Trucount analyses with flow cytometry. A set of 50 different serum cytokines, chemokines and factors of angiogenesis reflecting immune cell activation and regulation of immune response will be measured using Luminex-based multiplex assays (Table 1).

**Table 1:** Serum cytokines, chemokines and factors of angiogenesis analyzed in ChilSFree

Cytokines		Synonym	Chemokines		Synonym
TH1 response	IFN-γ		CCL chemokines	CCL2	MCP-1
	IL-2			CCL3	MIP1a
	IL-12			CCL4	MIP-1b
	G-SCF				
	GM-CSF			CCL5	RANTES
	TNF-α			CCL7	MCP-3
TH2 response	IL-4			CCL11	Eotaxin
	IL-5			CCL27	CTACK
	IL-10		CXCL chemokines	CXCL1	Gro-a
	IL13			CXCL8	IL-8
TH9 responses	IL-9			CXCL9	MIG
TH17 response	IL-17			CXCL10	IP-10
	IL-23			CXCL12	SDF-1α
polyfunctional	IL-1a		*Growth factors*	M-CSF	
	IL-1b			SCF	
	IL-1RA			SCGF	
	IL-3			PDGF	
	IL-6			HGF	
	IL-18			FGF-b	
	LIF			MIF	
*Angiogenic factors*	Ang-2	angiopoietin-2	*Soluble surface molecules*	sCD25	IL-2Rα
	VEGF			ICAM-1	
	PECAM-1	sCD31		VCAM	
	Leptin			TRAIL	
				Follistatin	

### Clinical data collection

Clinical parameters to be documented include demographic data such as age, sex, weight, height and primary disease of the patients, transplant associated information such as the indication for transplantation, donor-recipient relation (living related donation or cadaveric transplantation, blood group compatibility), type of graft (split or full-size) and surgical as well as non-surgical complications. Information on infections is collected both as a documentation of the EBV- and CMV serostatus at transplantation, and as documentation of viral and bacterial infections in the course of the study. Immunosuppression will be documented with regards to type of IS medication, dose and trough level at the study visits and changes of IS medication with the respective reasons for changing the therapy. In cases of liver biopsy, presence of acute cellular rejection is noted both as a binary choice (rejection vs no rejection) as well as on an ordinal scale with grading according to the RAI score. A full list of clinical parameters to be documented is given in Table 2.

**Table 2:** Clinical parameters recorded in CHilSFree

Basic patient data	
Age	
Sex	
Weight & height at transplantation	
Primary diagnosis	
Comorbidities before transplantation	
Concomitant medication at time of transplantation	
Biochemistry and hematology before transplantation	
Transplant-related data	
Indication for transplantation	
Type (split / whole liver) and weight of graft	
Living related transplantation	
Donor information: age, gender, cause of donor death	
Blood group compatibility	
HLA status recipient	
Infection related data	
CMV and EBV status of donor and recipient	
EBV/CMV infection	
Immunosuppression	
Use of basiliximab for induction; IS drugs and trough levels at every visit, changes in IS drugs and reason for changes	
Complications	
Standardized documentation of portal vein stenosis, portal vein thrombosis, arterial stenosis, artherial thrombosis, sepsis, hepatic vein stenosis, biliary obstruction, bile leak, wound infection, prolonged ventilation, inotrope support, PTLD; additional documentation of “other” complications	
Follow-up visits	
Weight, height, biochemistry, hematology, virology (viral load CMV / EBV), changes in immunosuppressive medication, immunosuppressive drugs (dose and trough level), additional medication, additional comments	
Biopsy visits	
Results of liver biopsy (rejection none, mild, moderate, severe and RAI score)	

### Data management and documentation

For documentation of clinical parameters, an electronic case report form (eCRF) using the Marvin software (xclinical) was created. Parameters to be documented in the eCRF were compiled using input from all participating centers. After creation and testing of the eCRF, a pilot phase was initiated with data entry from all participating centers, followed by a focus group that discussed feasibility of documentation in everyday clinical practice, unambiguity of data entries and missing items that were deemed to be important. The eCRF was amended accordingly before definitive data capture commenced. Special note in the eCRF amendment was put on the documentation of rejection episodes as this information was regarded as vital for later data analysis. Monitoring of data entries into the eCRF is performed in a stepwise fashion. Participating centers are responsible for correctness of data entry locally and for compliance of their data with the local source data. In addition, a central data monitoring will be provided by the organizing center that will focus on plausibility of data entries and conformity of data documentation between the different participating centers. Queries are sent out centrally to the respective centers as well and will then be answered locally to improve data quality.

### Data analysis

Sample size calculation was originally performed based on the a priori hypothesis that two subgroups with differing risks of rejection (20% for the low risk group, 60% for the high risk group, frequency ratio 10:1, overall rejection risk of 25%) can be identified using immune monitoring. Taking into account 5% loss to follow up, 220 participants with available biosamples would be necessary to assess this hypothesis with 90% power and a two-sided alpha of 5% (chi squared test) in a confirmatory manner.

However, the main part of the ChilSFree study is designed to be hypothesis generating and focuses therefore on exploratory analyses. This includes the definition of post-transplant trajectories of immune response and the evaluation of the role of these trajectories as predictors for the presence or absence of tolerance. We will use flexible joint modelling approaches [14] in combination with suitable feature selection algorithms for high-dimensional data as e.g. boosting [15] or knowledge-based latent classes [16] to build predictive models for time to establish outcomes. Dynamic prediction methods will then be used to assess the predictive potential of the respective models [17].

### Description of the study population

**Fig. 2:**
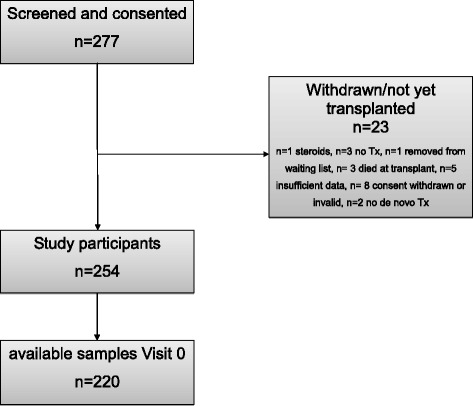
Flowchart of patient enrolment

Enrolment for the ChilsFree Study started in December 2012. To date, *n* = 277 children have been screened and provided informed consent for the study. Twenty three children had to be excluded from the study for various reasons (Fig. 2), leaving 254 study participants (56% male, 44% female). Samples for Visit 0 (pre-transplant) are available from 220 patients as requested in the sample size calculation. Participants’ age ranged from 0.2 to 18.0 (median 2.5) years. Biliary atresia was the most frequent underlying diagnosis (*n* = 101, 39.8%, Table 3). Living related transplantation was carried out in *n* = 72 (28.3%) cases. The vast majority of transplantations were ABO compatible, with only *n* = 13 being ABO incompatible (5.1%).

**Table 3:** Baseline characteristics of the study population

Subjects	n (%) / median (range)
Sex	
Male	142 (56%)
Female	112 (44%)
Age	2.5 years (0.2–18.0)
Diagnosis	
Biliary atresia	101 (39.8%)
Malignant liver disease	24 (9.4%)
Metabolic liver disease	21 (8.3%)
Acute liver failure	10 (3.9%)
PSC	7 (2.8%)
PFIC	12 (4.7%)
Alagille syndrome	11 (4.3%)
Cystic fibrosis	11 (4.3%)
Wilson’s disease	4 (1.6%)
Congenital hepatic fibrosis	3 (1.2%)
Toxic liver damage	1 (0.4%)
Other	49 (19.3%)
Type of graft	
Living related transplantation	73 (28.7%)
Cadaveric transplantation	181 (71.3%)
ABO compatibility	
ABO compatible	241 (94.9%)
ABO incompatible	13 (5.1%)

*Abbreviations: PSC* primary sclerosing cholangitis, *PFIC* progressive familial intrahepatic cholestasis

## Discussion

We propose an observational longitudinal study on immune monitoring in children after liver transplantation. Children will be followed from pre transplantation up to 12 months after transplantation and will receive regular extensive immune monitoring assessments. Findings will be linked to clinical changes, especially the occurrence of biopsy proven rejection (BPAR) and infections, with the aim to identify predictors of rejection or infection and delineate candidates for future immune monitoring and guidance of immunosuppressive therapy in clinical practice.

While there is a considerable number of studies that have investigated changes in the immune system after solid organ transplantation, notably with regards to T cell activation and proliferation, their use for application in children is very limited. Most published studies target adults [3–7, 13, 18–22] and predominantly investigate patients after kidney transplantation not liver transplantation [4, 5, 13, 19, 20, 23]. In pediatric liver transplantation a number of differences from adult liver transplantation might affect immune monitoring results warranting a specific pediatric approach. The distribution of primary diseases leading to liver transplantation is different in children where there are hardly any viral hepatitis patients, no alcoholic cirrhosis patients and hardly any NASH/NAFLD patients among the transplant population. Biliary atresia remains the major cause for pediatric liver transplantation ranging from 40 to 80% in published case series [24]. Other important underlying diagnoses include acute liver failure, autoimmune liver disease, alpha-1-antitrypsin deficiency, metabolic liver disease and hepatoblastoma, all with shares between 5 and 8% [24], underlining the heterogeneity of the pediatric transplant population. It is easily conceivable that primary diagnoses as different as hepatoblastoma (with or without chemotherapy), autoimmune liver disease and biliary atresia might lead to very different immune layouts before transplantation, with uncertain impact on the reaction to immunosuppression after transplantation. In addition, age has been shown to have a major impact on circulating TH1 and TH2 cytokine levels that is irrespective of immunosuppressive therapy or rejection [9]. These findings underline why results from adult studies cannot be easily transferred to children. Any study on immune monitoring in children will have to take into account differences and characteristics that might result from age and development.

To our knowledge, all published pediatric studies are single-center studies with small patient numbers. Our study will benefit from a multicenter approach in order to obtain an increase in sample size that will permit differentiated analysis.

Our study proposes a longitudinal rather than a cross-sectional approach. In previous studies that focused on longitudinal evaluations of immune markers after solid organ transplantation the main differences that could be found between rejectors and non-rejectors were intra-individual changes in immune markers when compared to baseline. A study on 40 children with intensive follow-up until day 28 after transplantation described distinct trajectories of circulating cytokine levels [8]. Differences in early trajectories were associated with the occurrence of early BPAR. In another study on adult transplant recipients, a spike in TH1 cytokines around rejection episodes was observed [11]. Our longitudinal approach will allow for a better understanding of the early changes after transplantation anticipating the baseline state of innate and adaptive immune system before transplantation. Our evaluation will clarify whether there are specific reaction patterns in the immune system that can be recognized at an early time point and that have a predictive meaning for the further immune response of the patient towards the occurrence of rejection or infections at a later date. The analysis in our proposed study will incorporate rejections throughout the whole first year after transplantation. Rather than performing a step by step comparison or comparison to baseline, our statistical approach will focus on taking into account the entire trajectory of the different patterns of immune reaction from pre transplantation throughout the first year after transplantation.

Immune markers tested in previously published studies used various methods for assessing T cell activation and proliferation, immune cell numbers and circulating cytokines. As discussed above, an ideal monitoring tool would be easy to use in routine clinical care, yield rapid results that enable dose adjustments and a timely control of their effect and would be cost-effective. We have chosen immune cell numbers by Trucount analysis and circulating cytokines and chemokines as potential immune markers.

The host’s immune response against the graft is based on a combination of different immune cells [25]. The recognition of allo-antigens is based on an interaction of foreign donor major histocompatibility complex (MHC) I and II molecules with recipient CD8+ and CD4+ T cells. B cells, dendritic cells and natural killer (NK) cells all exist in inflammatory and anti-inflammatory subpopulations and are hence likely to contribute to allograft rejection [25]. Trucount analysis will facilitate monitoring the balance between pro- and anti-inflammatory subpopulations and uncover expansion of effector populations. Previous studies have shown that rather than absolute cell numbers it is the balance between pro- and anti-inflammatory immune phenotypes that determines outcome [5, 9]. In addition, the Trucount technology offers comparatively quick results. In view of the quantity of samples to be analyzed and the future applicability for clinical practice it appears necessary to restrict the analysis to quantification of different immune cells, but omit the technically more advanced and complicated analysis of e.g. T cell proliferation or activation used in other studies.

Soluble cytokine levels in the peripheral blood represent a compound measure of activation of different immune cells. One disadvantage of this technique is that peri-surgical stress, infection and ischemia-reperfusion injury might also contribute to circulating cytokines and therefore, represent confounding factors at the immediate early phase after transplantation, i.e. within 24 to 48 h [8]. However, previous studies have clearly linked individual peripheral cytokines to immune status and occurrence of rejection after transplantation [8–10, 26]. Thus, analysis in peripheral blood offers the advantage of easily obtainable material and with even small sample volumes of less than 2 ml, which is suitable for children, clinically relevant information can be obtained and provide an insight into the recipient’s immune status. Our proposed multiplex analysis allows the determination of blood levels of up to 50 different cytokines and chemokines in a minimal amount of plasma. This combines the advantage of a thorough and differentiated evaluation of immune markers incorporating both pro- and anti-inflammatory markers with adequate feasibility in a pediatric setting where available quantities of blood are often very small.

### Summary

In summary, we propose a prospective longitudinal multicenter cohort study on immune monitoring after pediatric liver transplantation. This study is designed to enhance and expand existing knowledge on the immune response to immunosuppression after liver transplantation in children. Identification of trajectories of immune markers over time will facilitate the understanding of different types of immune reaction that might lead to BPAR. Moreover, the study will help to understand whether these different immune reactions are consequences of primary disease, developmental differences, or of innate differences within the individual immune system. The overall aim of our study is the detection of immune markers that will enable us to guide immunosuppressive therapy in the future.

## Abbreviations

AUC: Area under the curve
BPAR: Biopsy-proven acute rejection
CSA: Cyclosporin A
eCRF: Electronic case report form
IS: Immunosuppression
MHC: Major histocompatibility complex
MMF: Mycophenolic acid
NK: Natural killer
Tx: Transplantation
